# Evaluation of Durability Performance for Chloride Ingress Considering Long-Term Aged GGBFS and FA Concrete and Analysis of the Relationship between Concrete Mixture Characteristic and Passed Charge Using Machine Learning Algorithm

**DOI:** 10.3390/ma16237459

**Published:** 2023-11-30

**Authors:** Yong-Sik Yoon, Seung-Jun Kwon, Kyong-Chul Kim, YoungSeok Kim, Kyung-Taek Koh, Won-Young Choi, Kwang-Mo Lim

**Affiliations:** 1 Korea Peninsula Infrastructure Special Committee, Korea Institute of Civil Engineering and Building Technology, Goyang 10223, Republic of Korea; humbleyys@kict.re.kr (Y.-S.Y.); kim6069@kict.re.kr (K.-C.K.); kimys@kict.re.kr (Y.K.); ktgo@kict.re.kr (K.-T.K.); 2 Department of Civil and Environmental Engineering, Hannam University, Daejeon 34430, Republic of Korea; jjnu98@hannam.ac.kr; 3 Department of Applied Artificial Intelligence, Sung Kyun Kwan University, Seoul 03063, Republic of Korea; tipunst@skku.edu

**Keywords:** ground granulated blast furnace slag, fly ash, passed charge, long-term aged concrete, machine learning

## Abstract

In this study, accelerated chloride diffusion tests are performed on ordinary Portland cement (OPC), ground granulated blast furnace slag (GGBFS), and fly ash (FA) concretes aged 4–6 years. Passed charge is evaluated according to ASTM-C-1202 for 12 mixtures, considering water–binder (W/B) ratios (0.37, 0.42, and 0.47), GGBFS replacement rates (0%, 30%, 50%), and FA replacement rates (0% and 30%). The effects of aged days on passed charge reduction behavior are quantified through repetitive regression analysis. Among existing machine learning (ML) models, linear, lasso, and ridge models are used to analyze the correlation of aged days and mix properties with passed charge. Passed charge analysis considering long-term age shows a significant variability decrease of passed charge by W/B ratio with increasing age and added admixtures (GGBFS and FA). Furthermore, the higher the water–binder ratio in GGBFS and FA concretes, the greater the decrease in passed charge due to aged days. The ML model-based regression analysis shows high correlation when compressive strength and independent variables are considered together. Future work includes a correlational analysis between mixture properties and chloride ingress durability performance using deep learning models based on the time series properties of evaluation data.

## 1. Introduction

Concrete offers several engineering benefits in addition to being a cost-effective building material. Globally, more than 5.5 billion tonnes of concrete are used every year [1]. However, if reinforced concrete (RC) structures are preserved in extreme environments or if their usage duration is increased, various deterioration problems arise. The most common deterioration phenomena in RC structures include chloride ingress, carbonation, freezing and thawing, alkali aggregate reactions, and sulphate-induced erosion [1,2,3]. Among them, chloride ingress is a deterioration phenomenon caused by the penetration of chloride ions in an RC structure, causing the internal reinforcements to corrode. Additionally, iron-melting due to chloride ingress is a steadily increasing problematic phenomenon. The damage by chloride ingress is tremendous, particularly in the case of urban roadside concrete structures [4,5,6]. The chloride ions infiltrated into the RC structures are divided into those fixed by cement hydrate and free chlorides present in the internal pore water. Particularly, free chlorides are the main cause of chloride ingress and lead to local corrosion of rebar. Initially, rebar corrosion caused by chloride ingress leads to aesthetic problems such as rust stains, but structural problems eventually develop, resulting in a lack of integrity between concrete and rebar when the amount of corrosion exceeds the critical chloride content [1,2,3,7,8].

Numerous studies in the field of materials, design, and engineering have attempted to control the deterioration caused by chloride ingress. Methods using industrial byproducts such as ground granulated blast furnace slag (GGBFS) and fly ash (FA) as alternatives to cement are known to be effective [3,9,10]. Furthermore, the use of admixtures as replacements for cement can reduce carbon emissions from cement production plants, thereby efficiently mitigating environmental problems [11]. When GGBFS is used as a binder in concrete, the engineering performance is improved owing to high fineness and its own latent hydraulic reactions that densify the internal pore structures. Particularly, the resistance for chloride ingress is effectively improved, because CSH gel in concrete with GGBFS is increased by latent hydraulic reactions, and increased CSH gel adsorbs free chloride ions [1,7]. By using FA, one of the main admixtures, as a binder, long-term strength and durability performance improvements owing to pozzolanic reactions can be anticipated. Moreover, such improvements in workability and reduction in bleeding can be expected in uncured conditions due to the ball-bearing effect of spherical particles [3,12].

International concrete structure design standards perform chloride ingress durability performance designs based on Fick’s second law [13,14,15]. In South Korea, conservative analytical results are derived from environmental coefficients and durability reduction factors [13]. Fick’s second law requires details on the apparent chloride diffusion coefficient, surface chloride content, initial chloride content, cover depth, and exposure period. Among these, the chloride diffusion coefficient represents the rate at which chloride ions enter concrete, and is known to have a dominant influence on the durability performance analysis results [13,16]. The chloride diffusion performance of concrete is evaluated through an electrical accelerated test or a long-term saltwater immersion experiment. Representative electrical accelerated test methods include ASTM C 1202 and NT BUILD 492 [17,18]. The methods are relatively simple and offer the advantage of a short test period; however, chlorine ion movement is facilitated electrically. Thus, performing a chloride ingress durability performance analysis on the accelerated test results causes a highly conservative analysis [16,19]. In long-term saltwater immersion tests, chloride diffusion behavior can be analyzed in real environments. However, a minimum test duration of several months is required, and the test method has the disadvantage of relative complexity [9,19,20]. Recently, the study has been conducted to compensate for the difference between electrical accelerated tests and long-term immersion tests [19]. The chloride diffusion behavior of concrete can be varied by changing the mix design. However, previously suggested chloride diffusion behavior models of concrete are limited to predicting chloride diffusion behavior by simply considering the water–binder (W/B) ratio or admixture replacement rate [13,14,15,21].

Currently, machine learning (ML) and deep learning (DL) algorithms are actively used in a variety of fields, and they can be used to devise new techniques for the performance prediction and evaluation of concrete quality, considering several variables [22,23,24,25,26,27,28]. In related research, DL algorithms offer the advantage of extracting and automating complex features, especially from high-level abstract concepts. One of the key elements of DL algorithms is learning the main algorithm using a massive amount of collected data. Therefore, excellent performance in data classification and prediction is anticipated [26,27,29,30]. Furthermore, DL algorithms can self-identify and adjust variables to complement the limitations of conventional models [24,27].

ML is used to analyze the relationship between independent and dependent variables using linear and nonlinear regression analyses [28,31]. Dealing with regression problems involves supervised learning, which is one of the most common ML methods. ML algorithms offer the advantages of simple models and less complex calculation processes as compared to DL algorithms [32,33].

In the field of concrete performance, most of the existing studies conducted using ML and DL algorithms are limited to predicting mechanical and durable performances based on specific aged days and mix design [22,23,24,28]. However, concrete is a time-dependent building material whose performance improves with aged days [34]. Hence, time-dependent performance-changing behaviors can be effectively analyzed using ML and DL models [24,35,36,37].

This study quantifies the evaluation results of passed charge and compressive strength for OPC, GGBFS, and FA concretes aged 4–6 years. The test results obtained before 4 years of age are quoted from existing studies [38,39]. Furthermore, the effects of an increase in aged days on the compressive strength and passed charge behavior are quantified. Several ML algorithms are used to quantify the correlations of mix properties and passed charge with compressive strength.

## 2. Fabrication of OPC, GGBFS, and FA Concretes and Evaluation of Mechanical and Durability Performances

### 2.1. Used Materials and Mix Design

In this study, three levels of W/B ratio (0.37, 0.42, and 0.47), three levels of GGBFS content (0%, 30%, and 50%), and two levels of FA content (0% and 30%) were used for the concrete mix. The W/B ratio and substitution ratio of admixtures (GGBFS and FA) were considered to quantitatively analyze their impact on strength and resistance of chloride ingress. The designed strengths (f_ck_) for each mixture were set at 41 MPa (W/B: 0.37), 38 MPa (W/B: 0.42), and 33 MPa (W/B: 0.47) based on the respective W/B ratio. The slump for all mixtures was set at 150 cm, and to achieve this, varying amounts of super plasticizer were applied to each mixture. Due to the differences in the unit weight of binders (cement, GGBFS, and FA), there were slight variations in the aggregate quantities in 12 types of mixtures. All concrete specimens were subjected to temperature-controlled water curing (20 °C) until the target aged day. The physical and chemical properties of the binders used are listed in Table 1, while the properties of the aggregates are presented in Table 2. Portland cement for this study was specified in KS L 4201, and regulated to show compressive strength exceeding 12.5 MPa (3 aged days), 22.5 MPa (7 aged days), and 42.5 MPa (28 aged days). Additionally, the mix properties of the considered 12 levels of concrete mixes are listed in Table 3 [38,39].

### 2.2. Method of Evaluating the Passed Charge

In this study, the durability performance test method proposed in ASTM C 1202 was used to evaluate the chloride ingress resistance of OPC, GGBFS, and FA concrete mixes. The test method is essentially known to evaluate the chloride ion penetration resistance of concrete specimens. The proposed empirical equation (Equation (1)) was used to obtain the accelerated chloride diffusion coefficient from the resulting value of the passed charge [40].

**Figure 1:**
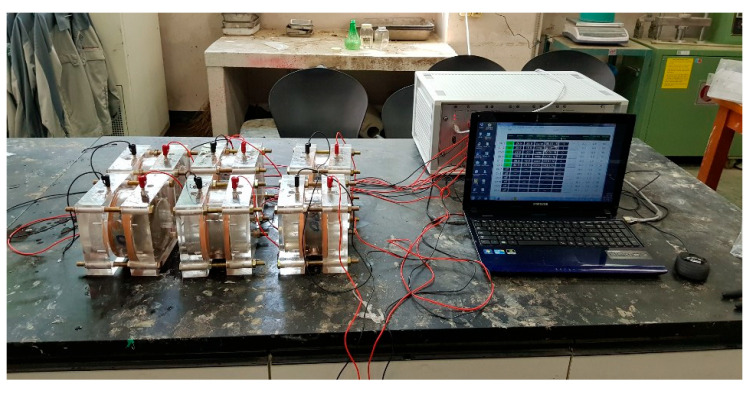
Photograph of ASTM C 1202.

For 6 h, a voltage of 60 V was supplied to an apparatus consisting of two cells and a disk specimen of thickness 5 cm, whose sides were coated with epoxy. Cell I (negative electrode) was filled with 3% sodium chloride (NaCl) aqueous solution, while Cell II (positive electrode) was filled with 0.3 M sodium hydroxide (NaOH) aqueous solution. The passed charge was measured and calculated every 30 min for a continuous supplied voltage using Equation (2). On each evaluation day, the amount of passed charge was calculated thrice, and the values were averaged to derive the final result. An image of the apparatus used to evaluate the passed charge is shown in Figure 1. ASTM C 1202 was used to evaluate the chloride diffusion resistance of concrete using the amount of passed charge based on the criteria listed in Table 4 [17].

(1) $$D=0.0.03\times 10^{-12}\times {\left(Q\right)}^{0.84}$$

where D denotes the accelerated chloride diffusion coefficient (m^2^ s^−1^) and Q denotes the passed charge (Coulomb).

(2) $$Q=900\left(I_{0}+2I_{30}+2I_{60}+\cdots +2I_{330}+I_{360}\right)$$

where Q. denotes the amount of passed charge (C) and $I_{x}$ denotes the current value (A) at the x-minute mark.

### 2.3. Method of Evaluating the Compressive Strength

The compressive strength was evaluated in accordance with KS F 2405 for OPC, GGBFS, and FA concretes aged 4–6 years [41]. Based on the compressive strength behavior for ~3 years, as evaluated by previous studies, an increase in the compressive strength behaviors with age was analyzed, considering ~2190 aged days or 6 years [38,39]. Similar to the amount of passed charge, discussed in Section 2.2, the compressive strength was evaluated thrice on each aged day for each concrete mix, and the average of the obtained values was used to analyze the results.

## 3. Results of Time-Dependent Mechanical and Durability Performance Evaluations for OPC, GGBFS, and FA Concretes

### 3.1. Results of Time-Dependent Compressive Strength Evaluation

**Figure 2:**
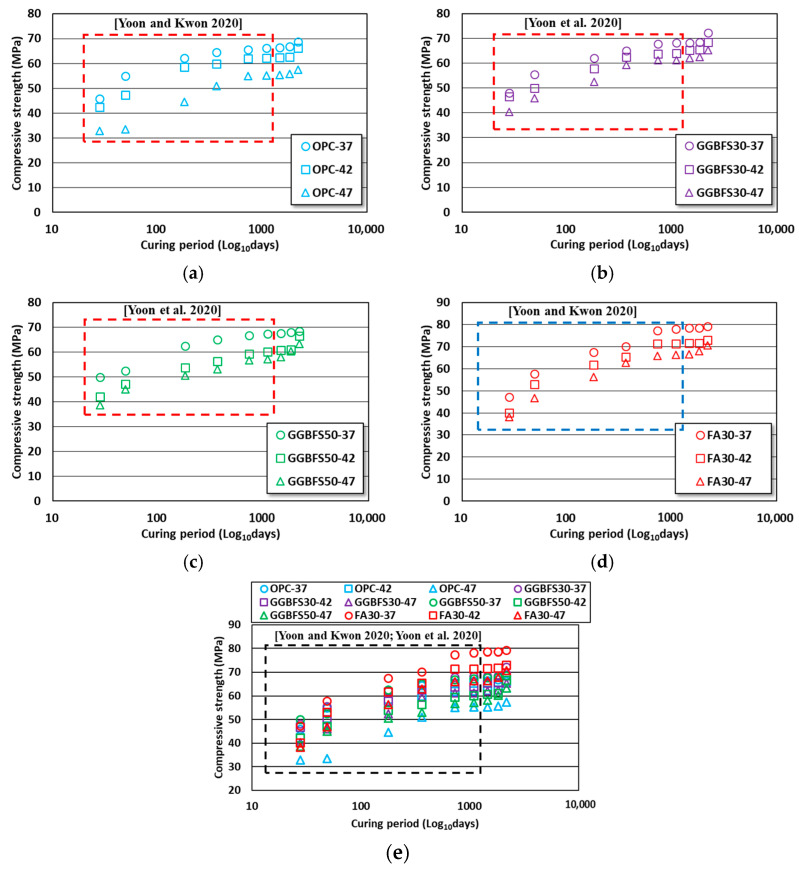
Compressive strength results of OPC, GGBFS, and FA concrete considering long-term age. (**a**) OPC concrete; (**b**) GGBFS30 concrete; (**c**) GGBFS50 concrete; (**d**) FA30 concrete; (**e**) all conditions [38,39].

The compressive strength evaluation results for the OPC, GGBFS, and FA concrete specimens aged 28–2190 days (6 years) are shown in Figure 2. The test results of the specimens aged 28 days to 3 years were used to analyze the time-dependent compressive strength behavior of each mix relative to the results of previous studies obtained with the same formulation [38,39].

**Figure 3:**
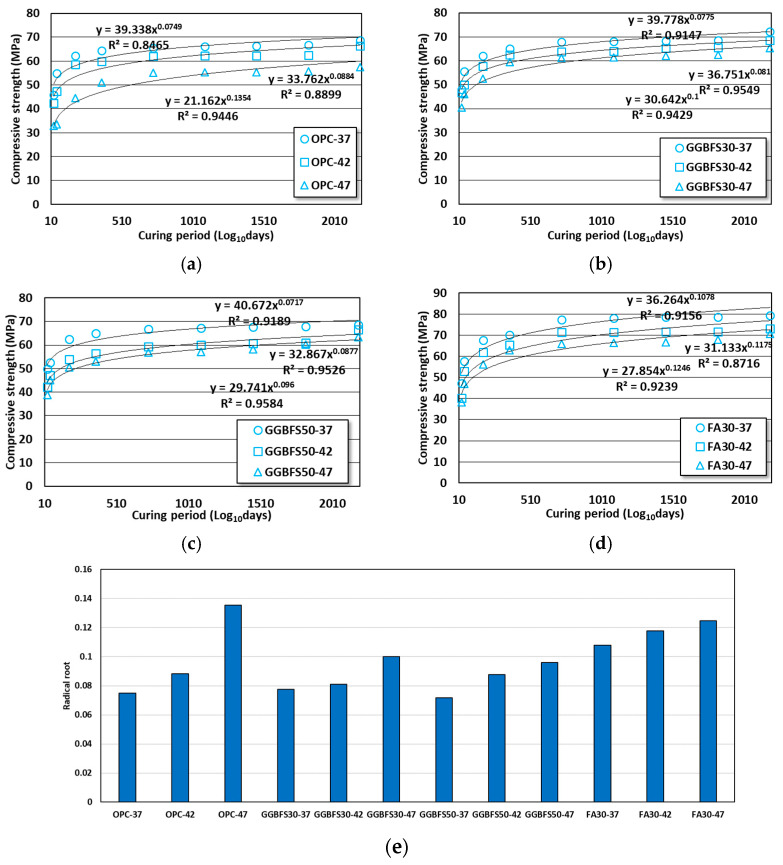
Effect of aged days on the compressive strength of OPC, GGBFS, and FA concretes. (**a**) OPC concrete; (**b**) GGBFS30 concrete; (**c**) GGBFS50 concrete; (**d**) FA30 concrete; (**e**) radical root of all conditions.

After 3 years, the last aged day in previous studies [38,39], no significant strength gains occurred in any mix. When the strength gains at 6 years were calculated based on the age day details of 3 years, the strength gains ranged from 3.9 to 6.7, 5.9 to 7.2, 1.8 to 11.1, and 1.4 to 6.5% for the OPC, GGBFS30, GGBFS50, and FA30 concretes, respectively. The GGBFS50 concrete showed the best long-term strength improvement, but no significant strength improvements were observed after 2 years of age in all concretes. It was assumed that most of the hydration reactions in all specimens ceased after two years, and in the case that admixtures were used, the difference in strength due to the W/B ratio appeared to decrease. Additionally, even in OPC concrete, with the low W/B ratio ensuring a sufficient amount of binder, a strength level similar to GGBFS concrete was observed. The effect of aged days on the compressive strength behavior was quantified by applying regression analysis in the radical root form, with aged days as the x-axis and compressive strength result as the y-axis. In this study, the magnitude of the square root represented the effect of aged days. The results of evaluating the effect of aged days on strength using the aforementioned method are shown in Figure 3.

Regression analysis of the aged day effect on the compressive strength behavior showed that the higher the W/B ratio, the larger the effect on all mixes. Particularly, aged days had the most effect on the OPC concrete, with a root mean square difference of 1.81 times between the W/B ratios of 37% and 47%. For the GGBFS concrete, the effect was at a similar level as the replacement rate increased. The values of FA concrete were between those of the OPC and GGBFS concretes. Even in OPC concrete, the specimen with a lower W/B ratio (0.37) appeared to be less influenced by the curing period. In the specimens containing GGBFS and FA, higher strengths compared to OPC concrete were showed from the early aged days. Consequently, even in concrete with a relatively low binder content (W/B ratio: 0.47), the impact of aged days on strength was at a low level. In the case of substituting cement with GGBFS or FA, reducing the amount of cement may cause a reduction of strength. However, a decrease in the amount of cement increases the free water available for reacting with the cement, promoting reactions of C_3_S and C_4_AF, which could lead to strength recovery. This phenomenon, termed the dilution effect, was substantiated in prior research [42]. Moreover, it was thought that the use of super plasticizer decreased the amount of water for concrete mixing, contributing to achieving initial strength more effectively.

### 3.2. Results of Time-Dependent Passed Charge Evaluation

**Figure 4:**
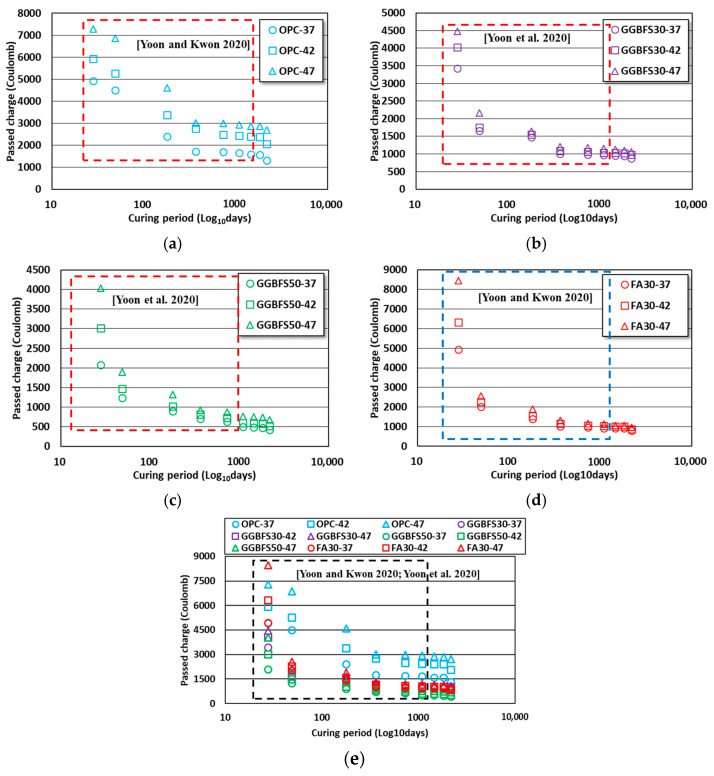
Passed charge results of OPC, GGBFS, and FA concrete considering long-term age. (**a**) OPC concrete; (**b**) GGBFS30 concrete; (**c**) GGBFS50 concrete; (**d**) FA30 concrete; (**e**) all conditions [38,39].

In accordance with ASTM C 1202, the passed charge evaluation results for the OPC, GGBFS, and FA concretes aged 4–6 years are shown in Figure 4. As discussed in Section 3.1, the test results of the specimens aged 28 days to 3 years were analyzed for time-dependent decreasing passed charge behaviors relative to the results of previous studies, for the same formulations [38,39].

Based on the last aged days of the previous studies, the rate of passed charge decrease for age days of 6 years was assessed based on the age days of 3 years, and the results were 7.9 to 20.7, 5.3 to 9.7, 10.0 to 14.7, and 13.6 to 16.5% for the OPC, GGBFS30, GGBFS50, and FA30 concretes, respectively. Comparatively, OPC concrete showed a greater passed charge decrease than the others. However, the decreased rate in other concretes was underestimated because a major decline had already occurred before the age of 3 years. In concrete mixed with admixtures such as GGBFS and FA, the passed charge variations among the 3 levels of W/B ratio decreased as age increased. In the case of OPC concrete, the variation by W/B ratio was relatively large, even at the final age of 6 years. Moreover, the high Blaine in GGBFS resulted in a packing effect, enhancing the durability performance of specimens containing GGBFS from the early aged days.

**Figure 5:**
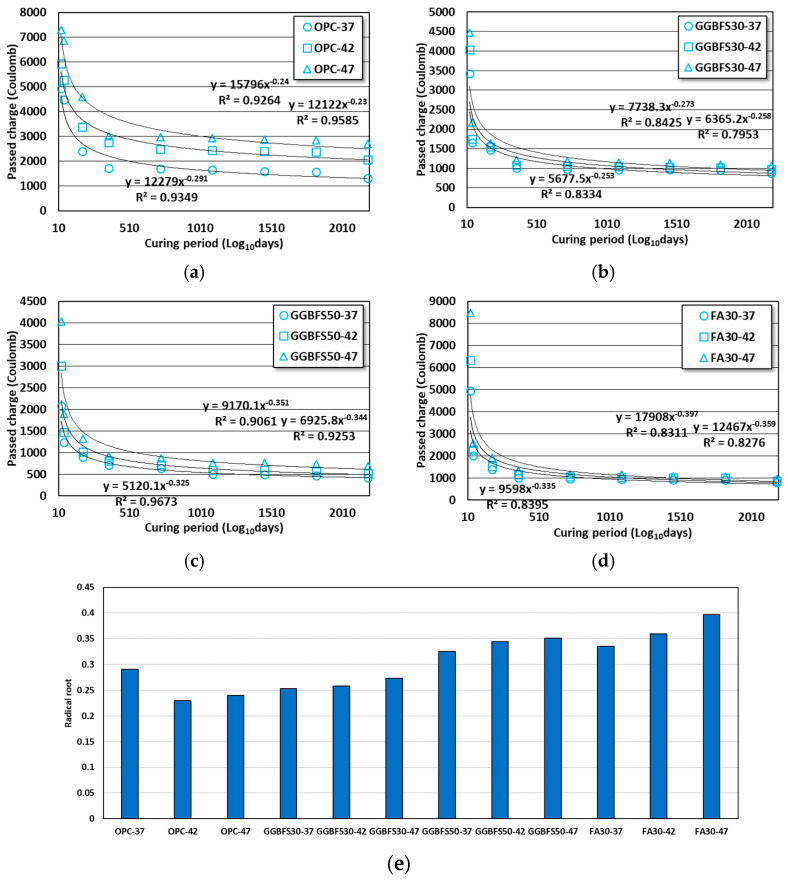
Effect of aged days to passed charge in OPC, GGBFS, and FA concrete. (**a**) OPC concrete; (**b**) GGBFS30 concrete; (**c**) GGBFS50 concrete; (**d**) FA30 concrete; (**e**) radical root of all conditions.

The design criteria for chloride ingress resistance performance in different countries consider the extent to which the behavior of the chloride ions penetrating into the concrete decreases over time. When the durability performance was analyzed without considering the diffusion behavior that was improved with dependence on time, the analysis results were very highly conservative, requiring crucial consideration [13,15,16]. The effect of aged days on passed charge was quantified by applying regression analysis considering the test results of the specimens aged 28 days to 6 years, with the x- and y-axis representing the aged days and passed charge, respectively. The analysis results are shown in Figure 5.

The effect of aged days on the passed charge was analyzed through regression analysis, and the higher the W/B ratio for the GGBFS and FA concretes, the greater the effect of aged days. In the specimens containing GGBFS, there was not a significant difference in the impact of curing period due to varying W/B ratios. However, in FA concrete, the effect of curing period was high, due to the relatively higher passed charge in 28 aged days. 

OPC concrete showed the highest value in the OPC-37 mix. Even when the formulation only used OPC as the binding material and the unit binder quantity was achieved through a low W/B ratio, a greater improvement in chloride ingress resistance performance with the age days was expected. For GGBFS and FA concretes, even when the W/B ratio was relatively high, their material properties of latent hydraulic and pozzolanic reactions were observed to significantly improve the chloride ingress durability performance over the long-term. The obtained results aligned with those observed in previous studies [3,7,9].

## 4. Correlation Analysis of Concrete Mix Properties with Mechanical and Durability Performances Using Machine Learning Algorithm

### 4.1. Machine Learning Algorithm for Regression Analysis

For this study, three multiple regression models were used to analyze the correlation between mix properties and concrete performance. The models included linear, ridge, and lasso regression. A multiple regression model was used to analyze the relationship between a dependent variable and one or more independent variables. This model was used as the multicollinearity showing that correlations between independent variables did not exist in the data used in this study [43]. The three models used in this study have the following characteristics. Linear regression is simple and interpretable, but might not handle multicollinearity or irrelevant predictors well. Ridge regression is useful for handling multicollinearity and preventing overfitting, but it does not perform variable selection and keeps all predictors in the model. Lasso regression performs both parameter shrinkage and variable selection, resulting in simpler and more interpretable models, but it might discard potentially useful predictors. 

As the number of explanatory variables in regression analysis increases, model selection difficulties arise, and with more variables included in the model, the complexity of the problems increases and interpretation becomes difficult. Methods of subset selection and shrinkage exist as statistical solutions to provide modeling convenience by arbitrary selection of variables.

Tibshirani proposed lasso regression as a shrinkage method for regression models, where a penalty equal to the square of the estimated regression coefficient was imposed [44]. Accordingly, most of the regression coefficients had a value of zero, thus enabling variable selection based on the correlation between the variables. The selection of an optimal subset was a discrete selection of variables based on the selection or exclusion of certain variables. Contrarily, lasso regression allowed for both a reduction in the regression coefficients and a continuous selection of variables. A unique advantage of lasso regression is that a simple model can be built based on the principle of sparsity, as the number of explanatory variables with zero estimated regression coefficient increases. Ridge regression, a typical penalized regression model, usually imposed an L2-norm penalty on the objective function of the least square method to improve the efficiency of estimating the regression coefficient. The lasso regression method was similar to the ridge regression method in that they both used penalization. However, as an L1-norm was used, most of the estimated coefficients were shrunken completely to zero. In both methods, the degree of penalization was controlled by a tuning parameter (λ), and increasing the value of the tuning parameter did not completely reduce the regression coefficient of the ridge regression to zero. Contrarily, lasso regression approached the minimization of the sum of the residual squares with the constraint (penalty) of the absolute value sum of the regression coefficients (L1-norm). Therefore, the signal from variables with high explanatory power was relatively amplified, while the weak signals (noise) from variables with weak correlation were rapidly reduced to zero and disappeared [44].

### 4.2. Building a Dataset for Predicting Concrete Performance Based on Machine Learning Algorithm

The dataset for regression analysis was constructed by considering the mix properties, compressive strength, and passed charge evaluation results of 12 formulations discussed in Section 3. The total data were divided into training and evaluation data in the ratio 8:2. According to previous research, there exists a correlation between compressive strength, chloride diffusivity, and porosity [45]. So, when constructing the dataset, the correlation between mix properties and chloride ingress durability performance was analyzed by considering three cases. First, the mix properties (including unit water, cement, FA, GGBFS, fine aggregate, and coarse aggregate quantities) and aged days were considered as independent variables, while passed charge was the dependent variable. Second, the mix properties and aged days were the independent variables, while compressive strength was set as the dependent variable. Last, the mix properties, aged days, and compressive strength were the independent variables, while passed charge was the dependent variable. The considered three cases are summarized in Table 5. 

### 4.3. Result of Correlation Analysis between Mix Properties and Concrete Performance Using a Machine Learning Model

**Figure 6:**
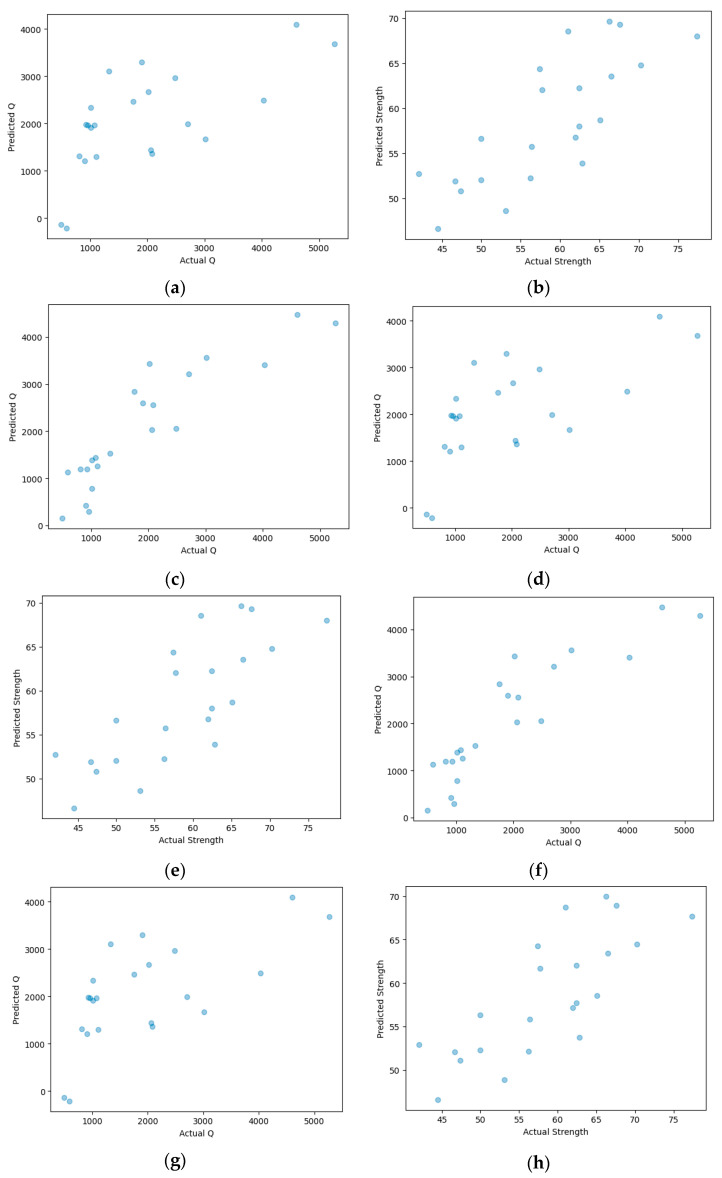

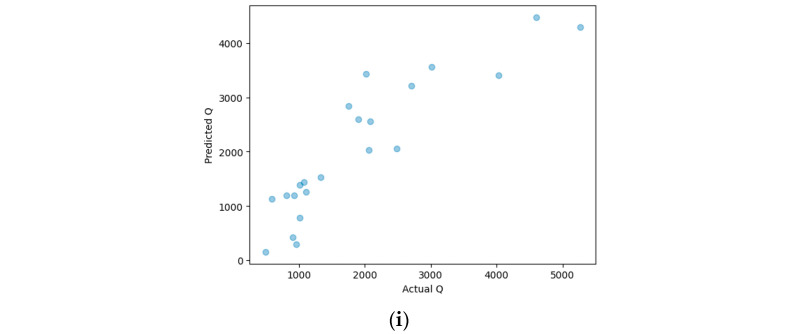
Results of linear, ridge, and lasso regression analyses considering three analytical conditions. (**a**) Linear analysis-case 1; (**b**) linear analysis-case 2; (**c**) linear analysis-case 3; (**d**) ridge analysis-case 1; (**e**) ridge analysis-case 2; (**f**) ridge analysis-case 3; (**g**) lasso analysis-case 1; (**h**) lasso analysis-case 2; (**i**) lasso analysis-case 3.

The correlations of concrete compression strength and passed charge with the mix properties were analyzed using the linear, lasso, and ridge regression models, considering the three cases listed in Table 5. The regression equations for each condition are listed in Table 6, and Figure 6 shows the relationship between the observed and predicted values for the test data in each condition.

The performance evaluation of the correlation of compressive strength and passed charge of concrete with the mix properties was verified by comparing the coefficient of determination (R^2^), which was an indicator of how well the independent variables explained the dependent variable in a regression model. The value ranged between 0 and 1, and higher values indicated better explanation [46]. The coefficient of determination was at a similar level between linear analysis and lasso analysis. Because those analysis methods are based on linear-oriented approaches, the levels were quite similar. It also suggested that both linear regression and Lasso regression were performing comparably in explaining the variance in the dependent variable using the given predictors. This could happen if the Lasso penalty was not aggressively eliminating variables, or if the dataset did not have many irrelevant predictors.

Little variation in R^2^ depending on the type of model used in the regression analysis was observed, and the coefficients of determination of the linear, lasso, and regression models all had a value of 0.789 when the compressive strength was considered as an independent variable for passed charge prediction. Thus, a relatively high correlation between the independent and dependent variables was obtained. Therefore, building a reliable model by considering the compressive strength while building a passed charge prediction model was possible.

## 5. Conclusions

In this study, the compressive strength and passed charge of 12 concrete mixes at 4 to 6 years of age were evaluated considering different admixture replacement rates. The effect of aged days on the mechanical and durability performance behavior was quantified by citing the results of previous studies that analyzed aged days. Furthermore, ML algorithms were used to analyze the correlation of compressive strength and passed charge with mix properties. The conclusions of this study are as follows.

1. Compressive strength analysis with increasing aged days showed that the strength increase was insignificant in every mix after 2 years of age. A regression analysis in a radical root form was performed on aged days and compressive strength to analyze the effect of aged days on compressive strength behavior. The effect of aged days on compressive strength increased with higher W/B ratios, and mixes with replaced admixtures were less affected by aged days than the OPC mix.
2. When the decrease in passed charge at 6 years of age was analyzed in comparison to 3 years of age, the last evaluation date of previous studies, the OPC mix showed a larger decrease than other mixes due to the fact that in other mixes, a significant decrease had already occurred before 3 years of age. The variability of passed charge due to W/B ratio decreased with increase in age when admixtures such as GGBFS and FA were added. A regression analysis with aged days as the x-axis and passed charge as the y-axis was performed to analyze the effect of aged days on passed charge behavior. For GGBFS and FA concretes, the effect of age increased as the W/B ratio increased. In the case of GGBFS and FA concretes, although the W/B ratio was relatively high, the material properties of latent hydraulic and pozzolanic reactions significantly improved the long-term chloride ingress durability performance.
3. When the correlation between mix properties and chloride ingress durability performance was analyzed by a multiple regression model in three cases, the best performance was observed when mix properties (unit quantity, unit cement quantity, FA quantity, GGBFS quantity, fine aggregate quantity, coarse aggregate quantity), aged days, and compressive strength were set as independent variables, while passed charge was the dependent variable. Thus, considering compressive strength in addition to mix properties and age can better explain the correlation between passed charge and mix properties. In the future, further analysis of the correlation between mix properties and chloride ingress durability performance through DL algorithms based on the time series characteristics of the data will be conducted.

## Figures and Tables

**Table 1:** Chemical properties of the cementitious material [38,39].

		Chemical Composition (Mass %)							Physical Properties	
	Items	SiO_2_	Al_2_O_3_	Fe_2_O_3_	CaO	MgO	SO_3_	Ig.loss	Specific Gravity (g cm^−3^)	Blaine (cm^2^ g^−1^)
Types										
OPC		21.96	5.27	3.44	63.41	2.13	1.96	0.79	3.16	3214
GGBFS		32.74	13.23	0.41	44.14	5.62	1.84	0.20	2.89	4340
FA		55.66	27.76	7.04	2.70	1.14	0.49	4.3	2.19	3621

**Table 2:** Physical properties of the fine and coarse aggregates [38,39].

	Items	G_max_ (mm)	Specific Gravity (g cm^−3^)	Absorption (%)	F.M.
Types					
Fine aggregate		-	2.58	1.01	2.90
Coarse aggregate		25	2.64	0.82	6.87

F.M.: fineness modulus.

**Table 3:** Mix properties of the considered concrete [38,39].

	W/B (%)	Unit Weight (kg m^−3^)						S.P (wt%)
		W	C	GGBFS	FA	Fine Agg.	Coarse Agg.	
OPC	37	168	454	0	0	767	952	1.10
	42	168	400	0	0	787	976	1.00
	47	168	357	0	0	838	960	0.95
GGBFS30	37	168	318	136	0	762	946	1.30
	42	168	280	120	0	783	972	1.10
	47	168	250	107	0	835	956	1.00
GGBFS50	37	168	227	227	0	760	943	1.40
	42	168	200	200	0	780	969	1.20
	47	168	179	179	0	832	853	1.10
FA30	37	168	318	0	136	745	952	1.40
	42	168	280	0	120	768	953	1.20
	47	168	250	0	107	820	939	1.00

W/B: water–binder ratio, W: water, C: cement, GGBFS: ground granulated blast furnace slag, FA: fly ash, S.P: super plasticizer (wt% of binder).

**Table 4:** Evaluation standard for passed charge of chloride ions [17].

Total Passed Charge (C)	Chloride Ion Permeability
>4000	High
2000–4000	Moderate
1000–2000	Low
100–1000	Very low
<100	Negligible

**Table 5:** Independent and dependent variables for the analysis of relationship between passed charge and characteristics of concrete mixture.

	Independent Variables	Dependent Variables
Case 1	Water, Cement, FA, GGBFS, Sand, Gravel	Passed Charge
Case 2	Water, Cement, FA, GGBFS, Sand, Gravel	Compressive Strength
Case 3	Water, Cement, FA, GGBFS, Sand, Gravel, Compressive Strength	Passed Charge

**Table 6:** Regression equations by linear, ridge, and lasso analyses.

Analysis Method	Regression Equation	R^2^-Train Dataset	R^2^-Test Dataset
Linear analysis	Q=−11944.7−1.05×Days−5.33E−15×Water+5.48×Cement−4.1×GGBFS−0.949×FA+15.731×Sand+1.13×Gravel	0.512	0.407
	Strength=52.719−0.009×Days−1.39E−17×Water+0.056×Cement+0.066×GGBFS+0.103×FA−0.051×Sand+0.016×Gravel	0.700	0.611
	Q=−2617.7+0.510×Days+1.137E−13×Water+15.437×Cement+7.528×GGBFS+19.234×FA+6.619×Sand+3.975×Gravel−176.977×Strength	0.877	0.789
Ridge analysis	Q=−11940.0−1.05×Days+0×Water+5.48×Cement−4.11×GGBFS−0.946×FA+15.727×Sand+1.134×Gravel	0.512	0.407
	Strength=52.752−0.009×Days+0×Water+0.056×Cement+0.066×GGBFS+0.103×FA−0.051×Sand+0.016×Gravel	0.700	0.611
	Q=−2608.1+0.509×Days+0×Water+15.428×Cement+7.517×GGBFS+19.219×FA+6.615×Sand+3.972×Gravel−176.91×Strength	0.877	0.789
Lasso analysis	Q=−11901−1.05×Days+0×Water+5.458×Cement−4.131×GGBFS−0.918×FA+15.696×Sand+1.128×Grave	0.512	0.408
	Strength=86.201+0.009×Days+0×Water+0.037×Cement+0.044×GGBFS+0.078×FA−0.077×Sand+0.0107×Gravel	0.699	0.601
	Q=−2540.2+0.510×Days+0×Water+15.393×Cement+7.478×GGBFS+19.174×FA+6.561×Sand+3.962×Gravel−176.94×Strength	0.877	0.789

Q is the passed charge (Coulomb), days is the age, water is the unit water quantity (kg m^−3^), cement is the unit cement quantity (kg m^−3^), GGBFS is the unit GGBFS quantity (kg m^−3^), FA is the unit FA quantity (kg m^−3^), sand is the unit fine aggregate quantity (kg m^−3^), gravel is the unit coarse aggregate quantity (kg m^−3^), and strength is the compressive strength (MPa).

## Data Availability

The data presented in this study are available on request from the corresponding author.
